# Supplementary material for: Multiplex flow magnetic tweezers reveal rare enzymatic events with single molecule precision
Source: Nat Commun. 2020 Sep 18;11:4714. doi: 10.1038/s41467-020-18456-y (PMC7501243; doi:10.1038/s41467-020-18456-y)
Supplement: Supplementary file 1 — Supplementary Information [file 41467_2020_18456_MOESM1_ESM.pdf]

Supplementary Information

Multiplex flow magnetic tweezers reveal rare enzymatic events with single molecule precision

Agarwal, et al.

Supplementary Figures

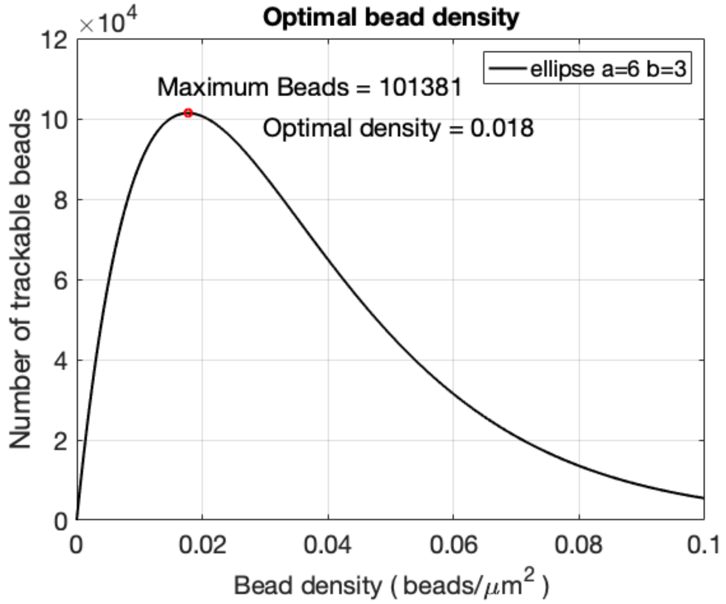

Supplementary Figure 1. Optimal bead density for random attachment sites. Bead density for maximum number of useful DNA-tethered beads was calculated assuming a random distribution of point-like particles on a 2D plane with ellipsoid profile according to De Vlaminck et al.[5]

$$\left(N_{beads} = A_{FOV}\int_{d_0}^{\infty}\omega(r)dr = A_{FOV}\rho e^{-\rho\pi d_0^2}\right) \quad (1)$$

$$N_{beads} = A_{FOV}\rho e^{-\rho\pi ab} \quad (2)$$

Where A_{FOV} is the area of the field of view (1.56e+07 μm^2), ρ is the bead density (beads/μm^2), a and b are the semi-major and semi-minor axes of the ellipse, respectively. More than 100,000 functional tethers could potentially be established for the given field.

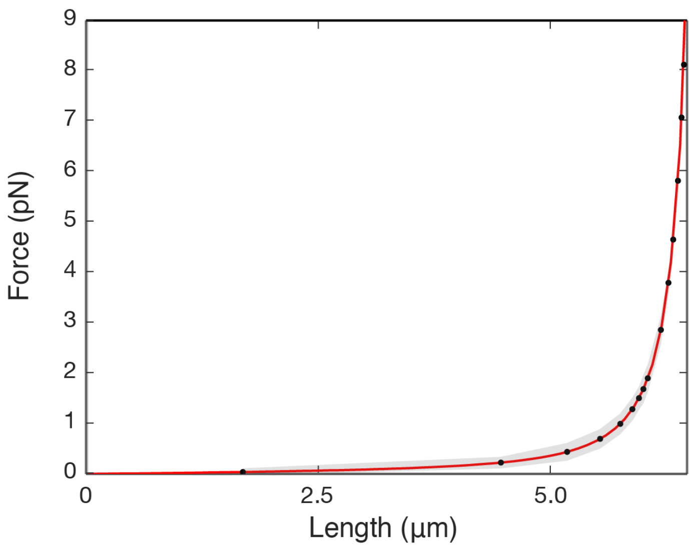

Supplementary Figure 2. Force extension behavior of single DNA molecules. Force versus length for DNA fit with the worm-like change model (red line). Black points represent the median (n = 1,244 molecules from one experiment). Gray area represents the standard error of the median.

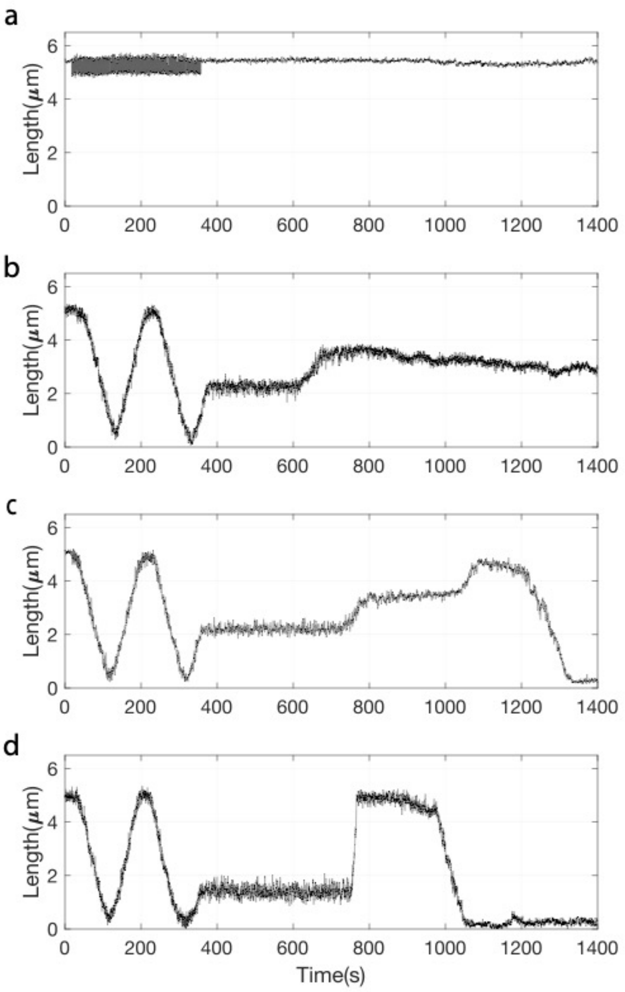

Supplementary Figure 3. Gyrase activity for different buffer conditions. A test for coilability was conducted at the beginning as follows: DNA was negatively supercoiled with 100 clockwise turns, uncoiled with 100 counterclockwise turns, positively supercoiled by 100 counterclockwise turns. After the coilability test, 40 clockwise turns were conducted leaving the DNA positively supercoiled prior to gyrase arrival. **a** Nicked molecules display no length changes due to magnetic rotation or introduction of gyrase. Distinct behaviors are observed for non-nicked molecules depending on the buffer conditions: **b** In the absence ATP, DNA length increases upon gyrase binding. **c** If ATP is supplied in the buffer with gyrase, DNA length increases in two steps. First, due to binding as displayed in b. Second, due to ATP-dependent relaxation of positive supercoils. The second step is followed by a pause and a decrease in length as gyrase

negatively supercoils the DNA. **d** If the flow cell is prewashed with ATP, the initial length increase is absent and only ATP-dependent relaxation of positive supercoils is observed. This is followed by a pause and then the length decreases as gyrase negatively supercoils the DNA. The rate of positive supercoil relaxation is approximately half in the absence of an ATP prewash. All experiments reported were conducted with an ATP prewash prior to introduction of gyrase into the flow cell.

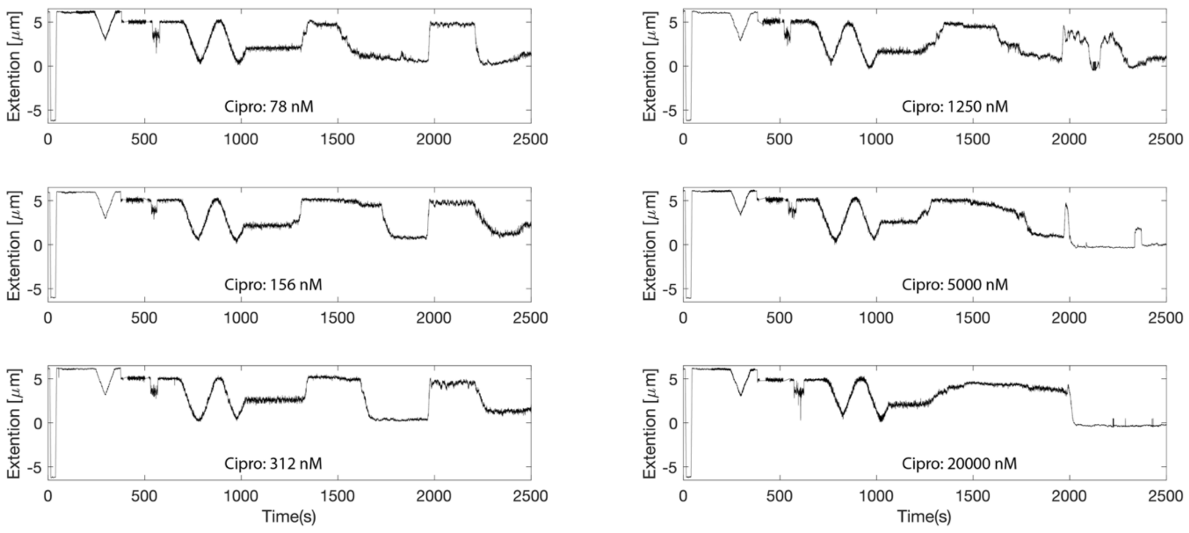

Supplementary Figure 4. Representative molecules for different ciprofloxacin concentrations. All experiments were performed under similar conditions with the exception of drug concentrations. Following the reversal to check for mobility, beads were checked for single tethering to the surface. Once the DNA was positively supercoiled, gyrase uncoils the excess positive coils and introduces negative coiling. Following the enzymatic activity, positive torque was applied using the external magnet @480 rpm for 4 minutes. Recovery of gyrase activity was then observed. It was observed that the velocity of gyrase activity was diminished with increasing drug concentration. At lower drug concentration, gyrase can still resolve the rapid positive coils introduced by the external magnet. As the drug concentration increases, gyrase barely introduces negative coils or resolves the positive coils respectively. Related to Fig. 5b & 5c.

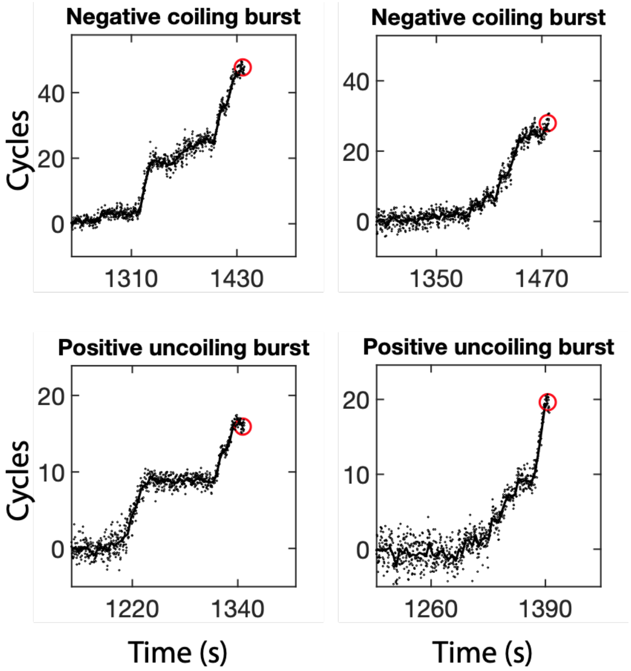

Supplementary Figure 5. Representative DNA breaks during normal gyrase activity bursts. Related to Fig. 5d.

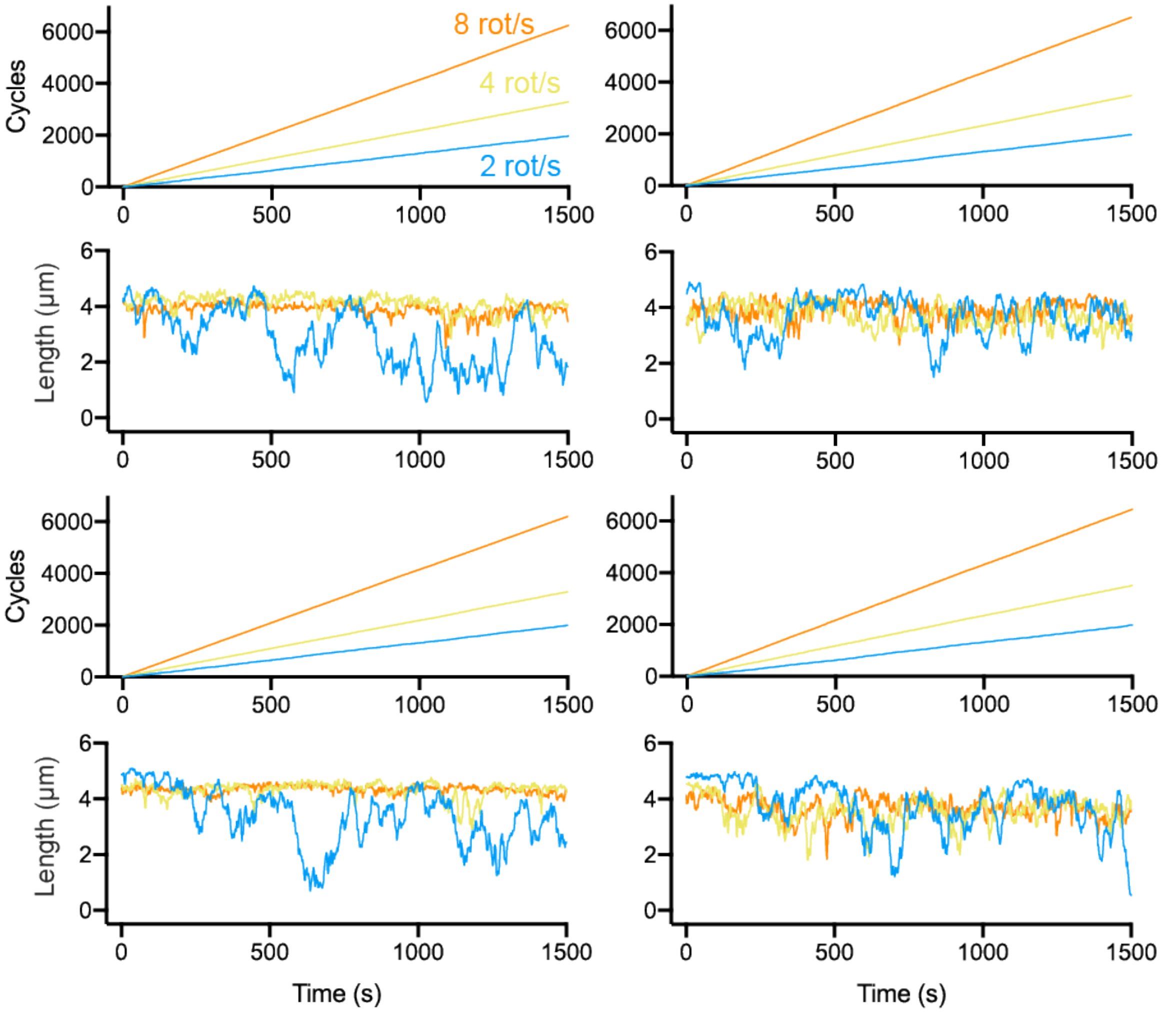

Supplementary Figure 6. Representative treadmilling molecules. Related to Fig. 5f,g.

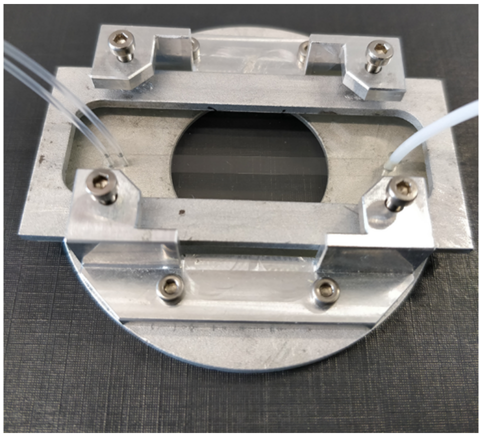

Supplementary Figure 7. Flow cell mounted in custom holder. Two inlets seen on the left, allow for fast buffer switching. One large outlet on the right is connected to a flow sensor used to maintain flowrate with a connected vacuum line. The central flow lane is 3 mm wide and 100 μm tall.

Supplementary References

1. Sitters, G. et al. Acoustic force spectroscopy. *Nat Methods* **12**, 47-50 (2015).
2. Berghuis, B.A., Kober, M., van Laar, T. & Dekker, N.H. High-throughput, high-force probing of DNA-protein interactions with magnetic tweezers. *Methods* **105**, 90-8 (2016).
3. Yang, D., Ward, A., Halvorsen, K. & Wong, W.P. Multiplexed single-molecule force spectroscopy using a centrifuge. *Nat Commun* **7**, 11026 (2016).
4. Strick, T.R., Allemand, J.F., Bensimon, D., Bensimon, A. & Croquette, V. The elasticity of a single supercoiled DNA molecule. *Science* **271**, 1835-7 (1996).
5. De Vlaminck, I. et al. Highly Parallel Magnetic Tweezers by Targeted DNA Tethering. *Nano Letters* **11**, 5489-5493 (2011).
