# Supplementary material for: Multiplex flow magnetic tweezers reveal rare enzymatic events with single molecule precision
Source: Nat Commun. 2020 Sep 18;11:4714. doi: 10.1038/s41467-020-18456-y (PMC7501243; doi:10.1038/s41467-020-18456-y)
Supplement: Supplementary file 3 — Description of Additional Supplementary Files [file 41467_2020_18456_MOESM3_ESM.pdf]

Description of Additional Supplementary Files

File Name: Supplementary Movie 1
Description: **Flow reversal in a typical FMT field of view**. The dimensions of the field are 5.2 mm x 3 mm where each pixel corresponds to 1.56 µm^2 after a binning of 2. The insets with yellow boundaries show 10x and 100x zoom of the vast field. The video shows a feature called flow reversal where flow is reversed at positive and negative 20 µl/min. This shows mobility of all the molecules. This field contains approximately 30,000 beads, out of which more than 16,000 beads are mobile molecules.

File Name: Supplementary Movie 2
Description: **Representative FMT experiment imaging gyrase dynamics**. A single molecule is shown on the left together with a series of plots on the right showing flow changes, magnet rotations, and bead tracking. After the sequence of flow changes and magnet rotations described in the text and displayed in Fig 4b, gyrase is introduced into the flow cell. Positive relaxation and negative introduction are observed when gyrase engages the DNA molecule. Following these events, the magnet is continuously rotated at 480 rpm for four minutes to constantly introduce positive supercoils. These are resolved in real-time by gyrase and the tether length remains constant. Finally, magnet rotation is stopped and gyrase negatively supercoils the DNA molecule resulting in rapid compaction at the end of the video. This video is sped up and slowed down at certain points to accentuate selected features of the trace.

File Name: Supplementary Movie 3
Description: **Representative FMT experiment imaging gyrase showing a DNA break during a burst of negative supercoil introduction.**
