# Supplementary material for: Multiplex flow magnetic tweezers reveal rare enzymatic events with single molecule precision
Source: Nat Commun. 2020 Sep 18;11:4714. doi: 10.1038/s41467-020-18456-y (PMC7501243; doi:10.1038/s41467-020-18456-y)
Supplement: Supplementary file 7 — Reporting Summary [file 41467_2020_18456_MOESM7_ESM.pdf]

nature research

Corresponding author(s): Karl E Duderstadt

Last updated by author(s): Aug 14, 2020

Reporting Summary

Nature Research wishes to improve the reproducibility of the work that we publish. This form provides structure for consistency and transparency in reporting. For further information on Nature Research policies, see Authors & Referees and the Editorial Policy Checklist.

Statistics

For all statistical analyses, confirm that the following items are present in the figure legend, table legend, main text, or Methods section.

n/a	Confirmed	
☐	☒	The exact sample size (*n*) for each experimental group/condition, given as a discrete number and unit of measurement
☐	☒	A statement on whether measurements were taken from distinct samples or whether the same sample was measured repeatedly
☐	☒	The statistical test(s) used AND whether they are one- or two-sided<br>*Only common tests should be described solely by name; describe more complex techniques in the Methods section.*
☒	☐	A description of all covariates tested
☐	☒	A description of any assumptions or corrections, such as tests of normality and adjustment for multiple comparisons
☐	☒	A full description of the statistical parameters including central tendency (e.g. means) or other basic estimates (e.g. regression coefficient) AND variation (e.g. standard deviation) or associated estimates of uncertainty (e.g. confidence intervals)
☒	☐	For null hypothesis testing, the test statistic (e.g. *F*, *t*, *r*) with confidence intervals, effect sizes, degrees of freedom and *P* value noted<br>*Give P values as exact values whenever suitable.*
☒	☐	For Bayesian analysis, information on the choice of priors and Markov chain Monte Carlo settings
☒	☐	For hierarchical and complex designs, identification of the appropriate level for tests and full reporting of outcomes
☒	☐	Estimates of effect sizes (e.g. Cohen's *d*, Pearson's *r*), indicating how they were calculated

Our web collection on statistics for biologists contains articles on many of the points above.

Software and code

Policy information about availability of computer code

Data collection	Norpix Streampix 6 was used for recording videos.<br>Elveflow software (ESI) version 3.02.04 was used for controlling the flow system.
Data analysis	Fiji (ImageJ) version 2.0.0-rc-71/1.52n was used for image analysis with custom written SciJava plugins in Java.<br>All code used in this study was deposited on GitHub in several repositories: https://github.com/duderstadt-lab/mars-core and https://github.com/duderstadt-lab/mars-fx, https://github.com/duderstadt-lab/mars-fmt and https://github.com/duderstadt-lab/fmt-scripts<br>The ForceCalculator found in https://github.com/duderstadt-lab/mars-core used was version 1.0.0-beta-4. Documentation is avaiable at https://github.com/duderstadt-lab/mars-docs<br>Matlab R2016b<br>Jupyter 4.4.0<br>Prism 8 (8.4.3 (471)) was used for generating plots.

For manuscripts utilizing custom algorithms or software that are central to the research but not yet described in published literature, software must be made available to editors/reviewers. We strongly encourage code deposition in a community repository (e.g. GitHub). See the Nature Research guidelines for submitting code & software for further information.

Data

Policy information about availability of data

All manuscripts must include a data availability statement. This statement should provide the following information, where applicable:

- Accession codes, unique identifiers, or web links for publicly available datasets
- A list of figures that have associated raw data
- A description of any restrictions on data availability

Raw videos for reproduction of the essential results are available at Zenodo with the following titles and links: Flow Magnetic Tweezers: Gyrase dynamics in absence of drug ciprofloxacin (https://doi.org/10.5281/zenodo.3981513), Flow Magnetic Tweezers: Gyrase dynamics in presence of 20 uM drug ciprofloxacin (https://doi.org/10.5281/zenodo.3981123), Flow Magnetic Tweezers: Gyrase dynamics under 3 different external torque conditions. Part 1/3 (https://doi.org/10.5281/

zenodo.3981531), Flow Magnetic Tweezers: Gyrase dynamics under 3 different external torque conditions. Part 2/3 (https://doi.org/10.5281/zenodo.3981542), Flow Magnetic Tweezers: Gyrase dynamics under 3 different external torque conditions. Part 3/3 (https://doi.org/10.5281/zenodo.3981545). Additional data and instructions available upon request.

Field-specific reporting

Please select the one below that is the best fit for your research. If you are not sure, read the appropriate sections before making your selection.

☒ Life sciences ☐ Behavioural & social sciences ☐ Ecological, evolutionary & environmental sciences

For a reference copy of the document with all sections, see nature.com/documents/nr-reporting-summary-flat.pdf

Life sciences study design

All studies must disclose on these points even when the disclosure is negative.

Sample size	The sample size in each individual experiment was determined by the number of individual molecules that satisfied a standard series of criteria that are outlined in figure 4a and the methods section. Specifically, molecules that did not reverse with flow or were not supercoilable were rejected.
Data exclusions	All individual molecules imaged in each experiment were tracked and processed. All observations were kept in each dataset. Then a series of tags were added to individual molecules based on a predetermined set of criteria outlined in the manuscript. Final plots excluded molecules that did not satisfy these criteria (i.e. Mobile beads, single DNA tether, non-nicked DNAs, etc.). This exclusion criteria was used for all force calibration experiments presented in Figure 3 and all gyrase experiments displayed in figures 4 and 5.
Replication	The force calibration experiments shown in figures 2 and 3 were replicated 8 times throughout a one year period. The gyrase experiment shown in figure 4 was replicated 23 times throughout a two year period. The gyrase experiments shown in figure 4 were all replicated more than 3 times during an 8 month period.
Randomization	This doesn't apply to our study. By default all molecules observed in individual experiments are randomized.
Blinding	Blinding was not possible because experiments were typically conducted by a single person performing all steps. Due to funding limitations and the complexity of the experiments conducted, investigators were not blind to the samples tested during experiments or analysis. This was not essential because analysis was conducted using automated classification and quantification.

Reporting for specific materials, systems and methods

We require information from authors about some types of materials, experimental systems and methods used in many studies. Here, indicate whether each material, system or method listed is relevant to your study. If you are not sure if a list item applies to your research, read the appropriate section before selecting a response.

Materials & experimental systems

n/a	Involved in the study	
☐	☒	Antibodies
☒	☐	Eukaryotic cell lines
☒	☐	Palaeontology
☒	☐	Animals and other organisms
☒	☐	Human research participants
☒	☐	Clinical data

Methods

n/a	Involved in the study	
☒	☐	ChIP-seq
☒	☐	Flow cytometry
☒	☐	MRI-based neuroimaging

Antibodies

Antibodies used	Anti-DIG fab fragments were used for coating micron sized beads. (Roche - several lots, catalog number 11093274910, from sheep)
Validation	The antibodies were covalently attached to beads and the beads were flowed through a chamber with single DNA molecules containing digoxigenin at one end. Flow was continued until the surface was saturated with beads attached to DNA molecules. Flow was then reversed and attached beads show a characteristic reversal that was twice the length of each individual DNA. Antibodies were not used for blotting or to determine specifically the amount of a protein or DNA. They were only used as a means to physically attach beads to individual DNAs that once formed were used for an entirely unrelated purpose.
